# Environmental impacts of cage culture in Lake Victoria: the case of Shirati Bay-Sota, Tanzania

**DOI:** 10.1186/s40064-015-1241-y

**Published:** 2015-09-04

**Authors:** Benedicto Boniphace Kashindye, P. Nsinda, R. Kayanda, G. W. Ngupula, C. A. Mashafi, C. N. Ezekiel

**Affiliations:** Tanzania Fisheries Research Institute (TAFIRI), P. O. Box 475, Mwanza, Tanzania; Tanzania Fisheries Research Institute (TAFIRI), P. O. Box 46, Sota, Shirati, Mara region, Tanzania

**Keywords:** Cage culture, Nutrient enrichment, Water quality, Waste food

## Abstract

The experimental cage culture was conducted at Shirati bay, Lake Victoria from February to August 2013, to investigate the impacts of the small scale cage culture on the environment. Three locations along the cages, at the intermediate and one in the offshore (control) were sampled for water quality parameters, phytoplankton and macro invertebrates. A notable increase in nutrient concentration was observed after the set of cages among the stations. However DO, pH, and water transparency showed no major changes and was within the recommended ranges. Cyanophytes an indicator of inorganic pollution dominated before and after the set of cages, an increase in phytoplankton numerical abundance was observed after stocking of fish in cages. In addition there was an increase in the invertebrate community especially bivalves and gastropods. In conclusion we found no consistent environmental change caused by cage culture, and therefore it can be allowed in Lake Victoria, Tanzania part, with close monitoring of its impacts.

## Background

Fish farming in cages though is the most common technology in marine waters, practiced mostly by developed countries (i.e. Norway, Germany, Netherlands) and most of the developing countries of South East Asia (i.e. China, Vietnam, Philippines, and Thailand). In most African countries it seems to be a new technology (FAO 2004). In East Africa for some extent, cage farming of Nile tilapia has been practiced for a while now by the Source of the Nile (S.O.N), a private company based in Uganda.

Declining catches of fish around Lake Victoria basin and growing demand for protein from fish has ultimately resulted into strengthening strategies of boosting aquaculture productions by the governments of the East African countries so as to fill the growing gap of productions from capture fisheries. The Lake Victoria being such large attracts cage farming unto it for increased fish yields. So far cage fish farming is not allowed by law in the Tanzanian waters of Lake Victoria on the fear of environmental pollution and other associated ecological effects. However, the demand for environmental impact assessment study for the positive consideration of cage farming in the Lake Victoria waters has been an issue of concern by the government of Tanzania for a decade now. The Association for Strengthening Agricultural Research in East and Central Africa (ASARECA) project in promoting the use of reservoirs and lakes to practice small scale cage culture in cooperation with Tanzania Fisheries Research Institute (TAFIRI), the practice is currently expanding.

Generally growing fish in cages may have negative environmental consequences. The possible consequences associated with cage culture farming include discharge of particulate and dissolved nutrients through uneaten waste feed, fecal matter, and excretory products (Masser 2008). And, this may negatively impact the environment by causing anoxic conditions in sediments (due to organic enrichments) underlying the cages, thus changing the invertebrates abundances and compositions (Ngupula and Kayanda 2010). In addition it may cause eutrophication due to nutrient enrichment of the water column (Ngupula et al. 2012). Furthermore farmed fish may escape and interact with other fish in the wild the results of which is the spread diseases and parasites. All these may result into ecological simplicity, and decrease in genetic diversity (due to genetic dilution) and increased mortality of the wild stocks (due to transferred diseases).

Since ASARECA project is critical in the development and promotion of cage aquaculture technology in Tanzania, a research component was designed and incorporated with the objective of checking on the feasibility of cage farming and the associated negative environmental issues.

Therefore, the present paper was aimed for testing the feasibility of cage farming in Lake Victoria waters and assesses some possible associated environmental consequences.

## Methods

### Study site

**Fig. 1:**
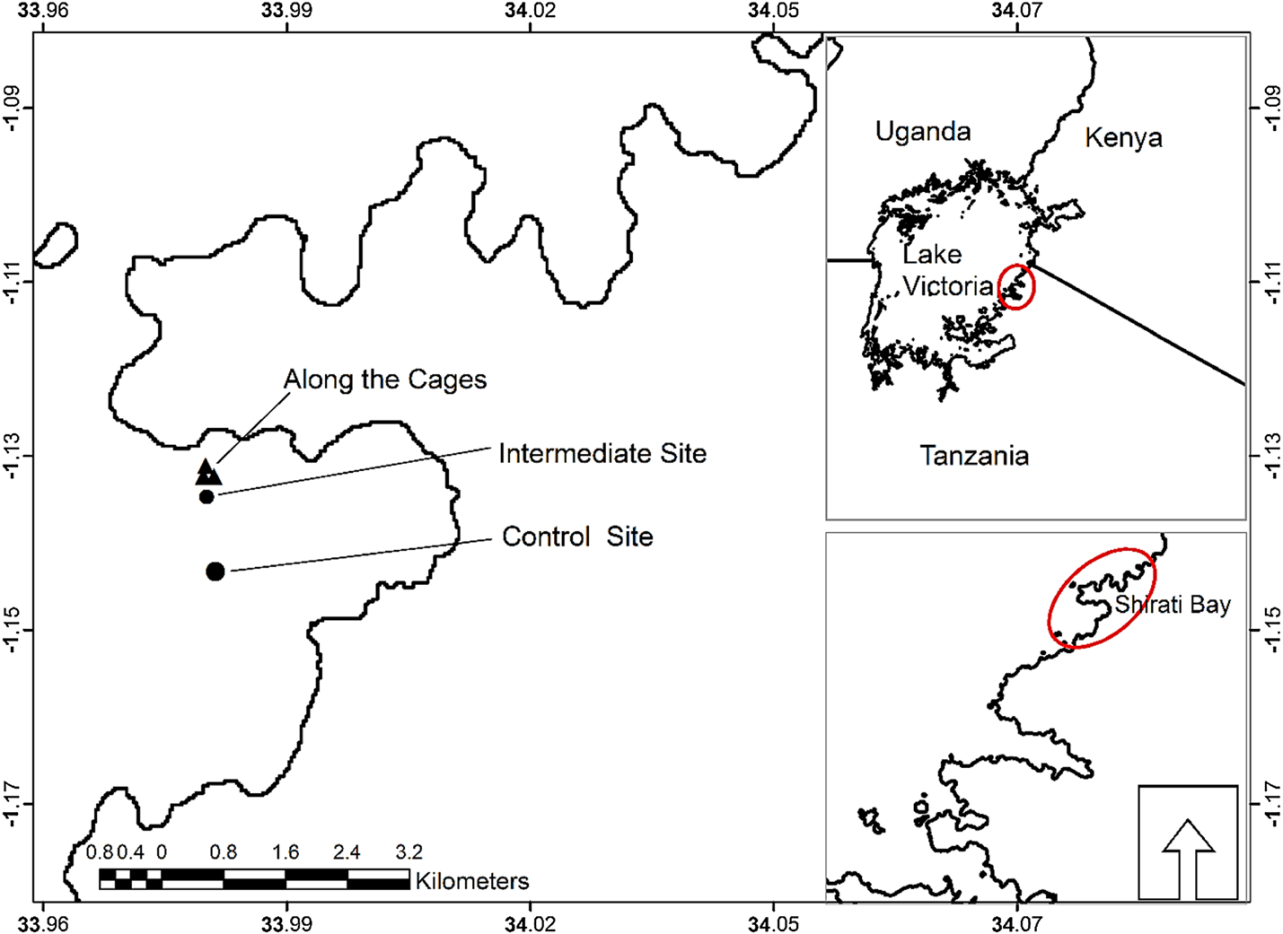
Map showing the sampling stations in Shirati bay, Lake Victoria

The sampling station (Shirati bay) where this study was carried out is shown in Fig. 1. Data was collected from February to August 2013. Shirati bay is approximated to have the surface area of 14 km^2^, the mean depth is 10 m. The deepest part of the bay is 15 m. Before deployment of cages for fish culture the environmental status of Shirati bay was assessed for its suitability for fish farming, including determining direction of the current. The current was flowing from South West to North East in the range of 4–6 knots in the morning and 7–10 knots in the afternoon. The cages were set at 1°8′3.78″S and 33°59′45.46″N near to the shore of, Shirati Bay, about 75 m offshore.

Nine cages of 2 m × 2 m × 2 m which were made of multifilament nets were set for Nile tilapia cage culture trials in February 2013. In order to detect the impact of fish cage culture in Shirati bay, three sampling locations close to each other along the cages, an intermediate site (50 m away from the cages) and a control site (located in south offshore waters 500 m away from the cages) were randomly selected and marked using a GARMIN Global Positioning System (GPS). The mean depth of the area where the cages were set was 7.0 ± 0.0 m, the intermediate station had the mean depth of 11.0 ± 0.0 m and the control had the mean depth of 12.0 ± 0.0 m. The cages were stocked with a total of 7041 fish of mean weights of 18.0 ± 2.1, 19.9 ± 14.7, and 18.5 ± 8.0 g in triplicates at stocking densities of 70, 100, and 130 fish/m^3^ respectively. The trials lasted for the period of 6 months. The fish were fed with TAF1 and Ugachick feeds both of 25 % crude protein. TAF1 feed is semi floating formulated feeds from locally available feeds, while Ugachick is commercial floating feeds imported from Uganda. The fish were fed at the feeding ration of 5 % per body weight three times a day at 1000, 1300 and 1600 h East African time.

### Water chemistry

Physicochemical parameters such as pH, DO, water temperature, Secchi depth and total depth were measured on weekly basis at 0900 h during the whole sampling period. In situ measurement of pH and DO was done using a calibrated portable Oxygen- pH probe (Model: 9024). pH and DO were measured for surface middle and bottom, the values obtained were averaged over the water column and calculated on monthly basis. Water transparency (m) was measured by a standard Secchi disk of 20 cm diameter, with quadrants painted in black and white. The Secchi depth was calculated as the average of the depth at disappearance and that of reappearance of the disk in water. Total depth (m) was measured by a graduated rope.

Water samples for the analysis of nutrients (Ammonia, Soluble Reactive Phosphorus, Total Nitrogen, Nitrites, Nitrates, Total phosphorus and Chlorophyll *a*) was taken on monthly basis for 3 months using a 1-l Van Dorn water sampler. Samples were collected for surface, mid, and bottom (about 0.4 m above the lake bottom and below the surface water) and mixed over the water column to make a composite sample per each sampling location. Samples were then preserved on ice pending analysis in the laboratory. Standard methods were used to analyze key nutrients, Total phosphorus (TP) was analyzed by Persulfate digestion method, Soluble reactive phosphorous, (SRP) by Ascorbic acid method, nitrate-nitrogen (NO_3_–N) by Cadmium reduction method, Nitrite Nitrogen by Colorimetric methods and Chlorophyll *a* concentrations by ethanol extraction method. Concentrations of these nutrients and Chlorophyll *a* were determined by spectrophotometry.

### Biological samples

Macro-invertebrates and phytoplankton diversity and abundance were sampled before stocking of fish and the start of feeding experiments in cages and 2 weeks before the end of the experiment.

Macro-invertebrate samples were obtained using a 384 cm^2^ Eckman grab sampler. Two hauls of the grab sampler was mixed to make one composite sample. A net of 500 µm was used to separate organisms from the sediments. The collected samples were preserved in 4 % formalin. In the laboratory the macro-invertebrates were identified and analyzed according to Brown (1994) and Mandahl-Barth (1958, 1973, 1988).

Plankton net of 10 µm mesh size was towed vertically three times from the bottom to the water surface to collect samples for phytoplankton species diversity at each sampling location. 100 ml concentrated sample was immediately preserved with 0.7 ml Lugol’s solution and after 1 h 2.5 ml were added to sample.

Samples for phytoplankton abundance were collected at the surface, middle and bottom by using a 1-l Van Dorn water sampler (the La MOTTE water sampler). The samples were immediately fixed as for diversity samples and placed in the dark cool box before analysis.

In the laboratory samples for diversity were examined using an inverted microscope at 400× magnification. Identification of phytoplankton species was done by using the available keys (Mosille 1984; John et al. 2002).

Samples for phytoplankton numerical abundance were sedimented for 48 h. A 20 ml concentrated sample, which remains in the bottom, was homogenized and 2 ml of it was taken for observation under an inverted microscope at 400× magnification. Different species were counted as numbers of filaments and cells depending on the nature of the phytoplankton. At least 30 fields were mounted from one sample.

The phytoplankton numerical abundance was calculated by using the formula given in Greenberg and Clesceri (1992):

$$= \, \left( {\left( {{\text{C }}*{\text{ At }}*{\text{ v}}} \right) \, / \, \left( {{\text{Af }}*{\text{ F }}*{\text{ V}}} \right)} \right)/{\text{V}}_{ 1}$$

where C = number of organism counted, At = total area of bottom of settling chamber (mm^2^), v = volume of concentrated sample (20 ml), Af = area of field (mm^2^), F = number of fields counted, V = volume of sample observed (2 ml), V_1_ = Volume of the sedimented sample.

Shannon diversity index (H′) (Shannon and Weaver 1949) was used to estimate macro-invertebrates and phytoplankton species diversity as follows:

$$H = - \sum\limits_{j = 1}^{S} {p_{i} } \ln p_{i}$$

where (*p*_*i*_) is the proportion of species *i* relative to the total number of species, which is multiplied by the natural logarithm of this proportion (ln*p*_*i*_). The resulting product is summed across species, and multiplied by −1. Shannon’s equitability (*E*_*H*_) was calculated as described by Magurran (1988) as follows; Equitability (Evenness) = H/Ln S, where H = Diversity index, S = Natural logarithm of the number of taxa (S). Species richness was obtained by simply counting the number of species present.

## Results

### Water quality

Data relative to dissolved oxygen, pH and Secchi depth are reported on Table 1. There were small fluctuation of pH, DO and Secchi depth among the sampling stations. Initially before the start of experiment at Shirati bay, the along the cages site had the mean depth of 7.0 ± 0.0 m. After stocking fish the mean depth slightly increased to 7.1 ± 0.8 m showing no or less deposition of materials. The mean depth of the intermediate station was 11.0 ± 0.0 m before the start of experiment and 11.0 ± 1.4 m after the end of experiment. While the offshore water at the location selected as the control site maintained the mean depth of 12.0 ± 0.0 m before and after the start of the experiment.

**Table 1:** Mean dissolved oxygen, pH and Secchi depth (mean ± standard deviation) averaged on monthly basis

		pH			DO (mg/l)			Secchi depth (m)		
		Along the cages	Control	Intermediate	Along the cages	Control	Intermediate	Along the cages	Control	Intermediate
February	Before stocking	5.28 ± 0.22	8.81 ± 0.43	5.25 ± 0.07	7.86 ± 132	8.56 ± 0.39	8.00 ± 1.30	1.21 ± 0.18	1.4 ± 0.17	1.16 ± 0.14
March	After stocking	5.23 ± 0.27		5.21 ± 0.21	7.04 ± 0.77		7.33 ± 0.96	1.40 ± 0.36		1.46 ± 0.36
April		5.22 ± 0.16		6.05 ± 0.68	7.16 ± 0.51		7.39 ± 0.34	1.29 ± 0.19		1.38 ± 0.21
May		7.49 ± 0.90		6.61 ± 0.60	6.25 ± 0.43		6.65 ± 0.30	1.63 ± 0.27		1.67 ± 0.23
June		7.22 ± 0.53		7.17 ± 0.70	7.28 ± 0.54		7.74 ± 0.71	1.53 ± 0.29		1.40 ± 0.37
July		6.80 ± 0.32		6.51 ± 0.17	7.29 ± 0.20		7.51 ± 0.21	1.29 ± 0.27		1.33 ± 0.31
August		7.45 ± 0.62	8.89 ± 0.45	7.18 ± 0.59	6.90 ± 1.14	8.60 ± 0.29	7.05 ± 1.12	1.61 ± 0.40	1.57 ± 0.38	1.76 ± 0.43
Mean		6.57	8.89	6.45	6.99	8.60	7.28	1.46	1.57	1.50

Almost all nutrient values indicated an increasing trend after the setting of the cages in both sites (along the cages, intermediate and control) except for Chl *a*. However highest increase was noted along the cages (Table 2).

**Table 2:** The mean concentrations (Mean ± SD) of nutrients values sampled along the cages (n = 4), at the intermediate site and the control site before and after stocking of fish in cages

Duration	Month	Location	TN	Nitrate nitrogen	Nitrite nitrogen	TP	SRP	Ammonia nitrogen	Chl *a*
	(µg/l)								
Before stocking	February	AC	482.31 ± 3.1	70.8 ± 9.6		89.8 ± 3.7	11.62 ± 0.9	168.3 ± 22.0	7.4 ± 2.3
		Intermediate	475.07	72.88		86.88	11.09	176	12.8
		Control	736.6	19.3		131.3	119.3	21.3	40.16
After stocking	March	AC	693.2 ± 96.3	81.6 ± 10.9		94.4 ± 25.1	15.9 ± 17.7	140.5 ± 70.9	2 ± 9.9
		Intermediate	728.38	76.75		76.24	5.3	112.25	3.64
	August	AC	1034.5 ± 282.0	137.2 ± 26.6	4.25 ± 1.5	106.8 ± 24.5		365 ± 126.2	1.902 ± 0.04
		Intermediate	748.2	113	4	92		328	1.94
		Control	985.6	32.9	1.3	151.1	125.8	294.0	32.59

Values recorded along the cages are averaged along the sampling locations

*TN* Total Nitrogen, *TP* Total phosphorus, *SRP* Soluble reactive phosphorous, *Chl a* Chlorophyll *a*, *AC* along the cages

### Phytoplankton diversity and abundance

Cyanophytes dominates in terms of numerical abundance both before and after stocking of fish in cages (Table 3). For example before stocking of fish in cages this group contributed about 96.6 % along the cages and 97.3 % at the intermediate site. After stocking of fish in cages Cyanophytes increased along the cages (99.6 %), and slightly declined at the intermediate site (93.9 %). Cyanophyte species that were common were *Anabaena flos*-*aquae, Anabaena spirodes, Merismopedia glauca, Aphanothece nidulans* and *Microcystis flos aquae.*

**Table 3:** Phytoplankton abundance (individuals/l) recorded at Shirati bay before and after the set of net cages

Taxa	Before stocking fish in cages		After stocking fish in cages	
	Along the cages	Intermediate	Along the cages	Intermediate
Chlorophyceae	30,688,359.8 (0.9 %)	75,015,467.5 (1.2 %)	481,826.9 (0.05 %)	782,968.8 (0.5 %)
Bacillariophyceae	57,259,776.2 (1.7 %)	94,205,470.8 (1.5 %)	4,119,969.3 (0.40 %)	8,492,199.5 (5.6 %)
Cyanophyceae	3,247,559,634.7 (96.6 %)	6,118,703,481.5 (97.3)	1,020,780,887.2 (99.6 %)	141,898,028.8 (93.9 %)
Dinophyceae	25,319,335.6 (0.8 %)	0(0 %)	0(0 %)	0(0 %)
Xanthophyceae	401,522.4 (0.01 %)	0(0 %)	0(%)	0(0 %)

The second largest group in terms of abundance was the diatoms which were higher at the intermediate site (5.6 %) and declined along the cages after stocking of fish (Table 3). The dominant diatoms were represented by *Nitzschia acicularis* and *Synedra cunningtonii.* Chlorophytes were poorly represented in terms of abundance and their abundance declined after stocking of fish in cages. However they were the second important group in terms of species richness after cyanophytes (Tables 3, 4).

**Table 4:** Phytoplankton species encountered along the cages and at the intermediate site (X = phytoplankton encountered before stocking fish in cages, + = phytoplankton encountered after stocking fish in cages, and X+ = phytoplankton encountered before and after stocking fish in cages)

Chlorophyceae	Along the cages	Intermediate
*Ankistrodesmus falcatus*	X	X
*Ankistrodesmus setigera*	X	X
*Chordatella subsala* var. *citriformis*	X	
*Coelastrum cambricum* var. *nasutam*	X	X
*Coelastrum microporum*		+
*Coelastrum reticulatum*	X	
*Dimorphococcus lunatus*	X	
*Kirchneriella contorta*	X	X
*Oocystis lacustris*	X	X
*Oocystis solitaria*	X	X
*Pediastrum duplex*		X
*Pediastrum simplex *var. *microporum*	X	X
*Pediastrum simplex* var. *duodenarium*	X+	
*Pediastrum simplex *var. *radians*	+	
*Scenedesmus* sp.	X	
*Staurastrum gracile* var. *nyassae*	X	X
*Staurastrum limneticum*	X	
*Staurastrum* sp.	X	
Bacillariophyceae		
*Aulacoseira granulata*	+	+
*Coscinodiscus rudolfi*	+	
*Cyclotella kützingiana*	X	
*Navicula* sp.		+
*Nitzschia acicularis*	X+	X+
*Rhizosolenia victoriae*	+	+
*Surirella biseriata*	X	
*Surirella fullebornii* var. *elliptica*	X	
*Synedra cunningtonii*	X+	X+
*Synedra ulna*	X+	+
Cyanophyceae		
*Anabaena flos aquae*	X+	X+
*Anabaena spirodes*	X+	X+
*Anabaenopsis* sp.		
*Aphanothece nidulans*		X+
*Chroococcus limneticus*	X+	X
*Chroococcus* sp.		+
*Chroococcus turgidus*	X	+
*Coelosphaerium* sp.		X
*Cylindrospermopsis curvispora*		X
*Cylindrospermopsis philippinensis*		X
*Merismopedia glauca*	X	+
*Merismopedia tenuissima*	X	X
*Microcystis aeruginosa*	X	X
*Microcystis flos*-*aquae*	X+	X+
*Planktolyngbya circumcreta*	X+	X+
*Planktolyngbya contarta*	X	
*Planktolyngbya limnetica*	X+	X+
*Planktolyngbya mertensiana*	X	
*Planktolyngbya tallingii*	X+	X+
*Planktolyngbya undulata*	X	X
*Pseudoanabaena* spp.	X	X
*Romeria* sp.	X	
Dinophyceae		
*Glenodinium* sp.	X	
*Ceratium limneticus*	X	
Xanthophyceae		
*Ophiocytium cochleare*	X	

### Macro-invertebrate diversity and abundance

**Fig. 2:**
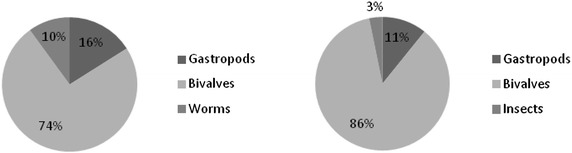
Percentage composition of benthic macro invertebrate groups sampled along the cages before and after stocking fish, Shirati bay

**Fig. 3:**
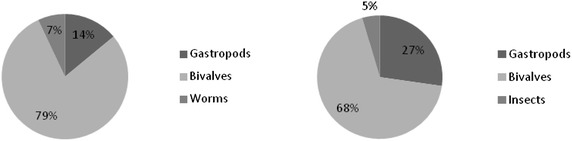
Percentage composition of benthic macro invertebrate sampled at the intermediate site before and after stocking fish at Shirati bay

**Fig. 4:**
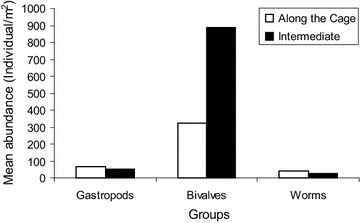
Mean abundance of different macro invertebrate groups before stocking fish in net cages at Shirati bay

The main groups of macroinvertebrates encountered at Shirati bay were mollusks, insects and worms. Before stocking of fish in cages in the inshore of Shirati bay bivalves were dominating with the mean of 107 ± 97.8 (74 %) individuals/m^2^ (Fig. 2). Gastropods made 16 % of the invertebrate community, which was about 104.1 ± 36.8 individual/m^2^. Both bivalves and gastropods constituted about 90 % of the total abundance of the benthic population. While worms made 10 % of the macro invertebrate abundance. A similar phenomenon was observed at the intermediate site, bivalves constituted 79 % (295 individuals/m^2^) followed by gastropods 14 % (52 individuals/m^2^) of the macro invertebrate abundance. Worms made 7 % (26 individuals/m^2^). Thus in both inshore (where the cages to be set) and intermediate, bivalves were dominating (Figs. 2, 3). However the highest abundance of bivalves was recorded at the intermediate site (Fig. 4).

**Fig. 5:**
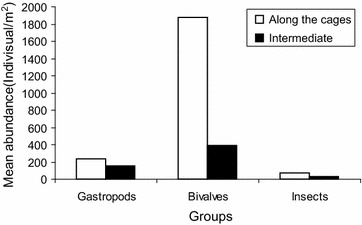
Mean abundance of different macro invertebrate groups after the start of cage farming activities at Shirati bay

After stocking fish in net cages bivalves community increased by 12 % in the inshore waters along the cages and 7 % at the intermediate site. During this time inshore waters along the cages had mean number of 468 (86 %) individuals/m^2^, gastropods had the mean number of 87.9 ± 46.0 (11 %) individuals/m^2^, and insects made 3 % of the invertebrate abundance (52.1 ± 21.3 individual/m^2^). The intermediate site in the offshore waters also was dominated by bivalves. However there was a decline in number of bivalves at the intermediate site by about 11 % after the set of cages. The mean abundance of bivalves was 97.6 ± 44.5 individuals/m^2^ (68 %), gastropods accounted for 27 % of the intermediate site in the offshore waters (78.1 ± 36.8 individuals/m^2^), with a decline of about 13 % after the set of cages. Insects were 5 % of the whole invertebrate community (26 individuals/m^2^) (Fig. 5).

### Benthic organism distribution by taxon

Initially before stocking fish in cages, gastropod of the viviparidae family *Bellamya costulata* was the only specie of gastropod found at Shirati bay. The highest abundance was found in the inshore waters where the cages were set (69 individuals/m^2^), the intermediate site had the least abundance (52 individuals/m^2^).

After stocking fish in cages species richness of gastropods increased (*B. costulata, Bellamya unicolor* and *Melanoides tuberculata)*. However *B. costulata* increased at both along the cages (121 ± 39.8 individuals/m^2^), and intermediate site (104 individuals/m^2^). *Melanoides tuberculata* (95 ± 15.0 individuals/m^2^) was the only species encountered along the cages. Higher abundance of *B. unicolor* was recorded at the intermediate site (52 individuals/m^2^) than those along cages (17 ± 15.0 individuals/m^2^).

Bivalves were more diverse and abundant before and after stocking fish in cages with *Sphaerium nyansa, Sphaerium stuhlmanni, Coelatura alluaudi and Coelatura monceti* dominating. Before stocking *S. nyansae* was higher in abundance at the intermediate site (651 individuals/m^2^) than in the inshore along the cages (95 individuals/m^2^). After stocking higher abundance was recorded in the inshore along the cages (1293 individuals/m^2^), while the intermediate had 78 individuals/m^2^. Before stocking higher abundance of *S. stuhlmanni* was recorded along the cages (139 individuals/m^2^) than the intermediate site (104 individuals/m^2^). After stocking there was an increase in abundance of *S. stuhlmanni* with 382 individuals/m^2^ along the cages and 156 individuals/m^2^ at the intermediate site. Before stocking *C. alluaudi* (35 individuals/m^2^) was only found along the cages in the inshore waters. After stocking *C. alluaudi* was found at both along the cages (78 individuals/m^2^) and the intermediate site (104 individuals/m^2^). Before stocking higher abundance of *C. monceti* were found at the intermediate site (130 individuals/m^2^) than the along the cage site (52 individuals/m^2^). After stocking *C.* *monceti* abundance increased along the cages to 121 individuals/m^2^, while the intermediate site remained the same (130 individuals/m^2^).

Insects of the class heptageniidae were only found along the cages after stocking fish in cages with 35 individuals/m^2^. While chaoborus larvae were also only found after stocking along the cages (35 individuals/m^2^) and the intermediate site (26 individuals/m^2^).

## Discussion

Fish farming in the lakes, is expected to produce waste with a high concentration of N and P released in solute form into the water column (Neofitou and Klaoudatos 2008). However, previous studies dealing with the analysis of plankton response to fish farming showed no significant difference between cages and control site (Pitta et al. 1999). Furthermore, several studies failed to establish a relationship between farm waste and phytoplankton growth, even when large inorganic nutrient inputs were recorded (Beveridge 1996). This study reports an increase in N and P in the inshore along the cages over the period of the cage trials. The offshore area which we considered as the control also experienced the same increase of N and P, although its increase was minimal compared to the inshore waters and the intermediate site. The increase in nutrient in the inshore along the cages may have been caused by the overall activities in the cages such as feeding. Other reason may be due to reduction in water movement caused by the presence of fish cages. This suggestion was verified by Iwama (1991), who reported reduction in water current velocity by 65 % inside the fish cages, mainly due to physical water obstruction by nets and organisms attached to them. In addition, other sources may have come from agricultural activities in the catchment which is reported to be the major contributor of N and P loading in the lake (Guildford and Hecky 2000; Scheren et al. 2000; Ngupula et al. 2012). After stocking of fish in cages the reported values of TN, Nitrate and Ammonia of this study were higher than previous findings in the same site. For instance results from TAFIRI 2005 technical reports show low values of TN (861.1 ± 176.0 µg/l), Nitrate (26.1 ± 9.6 µg/l) and Ammonia (157.6 ± 192.8 µg/l). However increase in nitrate concentrations is mostly linked to terrestrial run off (Talling and Talling 1965). Furthermore our findings for TP, SRP and Chlorophyll *a* were lower than the previous study at the same site as shown by the TAFIRI (2005) technical reports (TP = 141.2 ± 14.0 µg/l, Chlorophyll *a* = 36.4 ± 5.4 µg/l and SRP = 122.6 ± 4.6 µg/l). The Chlorophyll *a* values reported in this study and from the TAFIRI technical reports agree with Sitoki et al. (2010) who reported the chlorophyll *a* values of Lake Victoria rarely exceeds 30 and 50 µg/l in inshore and offshore respectively. Furthermore the chlorophyll *a* values we report on both stations lie well within the range of values given by Huszar et al. (2006) who obtained the mean values of 34.2 µg/l from 192 tropical lakes. From these findings it can be noted that the observed dynamics in N and P, Chlorophyll *a* mainly caused by seasonal land fluxes and phytoplankton uptake rather than the cage activities alone.

Fish farming enriches the water column with dissolved organic and inorganic nutrients and leads to a reduction in DO, both in the vicinity of the fish farm and at the site of remineralization of the waste products (Beveridge 1996; Mente et al. 2006). In this study the effect of nutrient discharge on DO was not pronounced. Our study report quite good dissolved oxygen level and Secchi disk reading throughout the study period, both along the cages, at the intermediate and control sites. These findings concur with Neofitou and Klaoudatos (2008), who found no effect of nutrient increase on DO in fish cages. The depth of the station also almost remained constant with minimal variations showing that there was no or less deposition of uneaten feeds.

The predominance of Cyanophytes at the Shirati bay may be attributed by the high concentrations of nutrients (Nitrate, ammonia and phosphorous). Eutrophic condition of the lake water has a tendency of allowing fewer species to grow faster and abundantly, this in turn permits a lower number of species to coexist (Moss 1998). This can explain the reasons of decline of species richness and diversity after establishment of cage culture activities, but the abundances of which were very high (Tables 3, 4, 5). The fact that most of the dissimilarities observed among the stations show changes in phytoplankton abundance rather than increase of typical species implies that there is relatively low intensity of disturbance caused by cage culture activities at the bay.

**Table 5:** Shannon diversity index, species richness and equitability (evenness) of phytoplankton species within the cage area and at the intermediate site recorded before and after stocking of fish in cages

	Within the cage area		Intermediate	
	Before	After	Before	After
Richness	41	15	27	17
Diversity index	1.268	0.476	1.435	1.818
Equitability	0.341	0.176	0.435	0.642

Generally, species richness decreased in both sites while diversity index decrease along the cage and increase at intermediate site. Equitability declined after stocking fish in cages within the cage area and increased at the intermediate site after stocking fish in cages

Aquatic macro invertebrates are used in pollution studies due to their typical abundance, relatively immobile and they can tolerate a wide range of pollution. The combination of macro invertebrate, phytoplankton and water quality studies can give a good explanation about the status of pollution of a particular water body. This study reports that even before stocking fish in net cages at Shirati bay, high abundance of mollusks (bivalves and gastropods) were recorded at both inshore (along the cages) and offshore (control), but the highest abundance being at the inshore (Table 6). After stocking fish in net cages an increase in bivalves and gastropods was noted at both the inshore and at the intermediate site, the highest being at the inshore along the cages (Table 7) . Unlike Apostolaki et al. (2007), who carried out a similar study in seaweeds farms reported a peak in macrofauna at the distance of 40 m from the cages. Like Apostolaki et al. (2007), species numbers showed little variability between the stations, and an increase in diversity index with increasing distance from the farm (Table 8). While the abundance of worms and insects were very low and even disappeared after stocking of fish in cages. The increase in gastropods and bivalves may be caused by the presence of organic materials that resulted from uneaten feeds and detritus materials which could have attracted both the gastropods and bivalves to obtain their source of food. Gastropods are reported to be the major browsers that feed on the organic cover of the bottom while bivalves are the filter feeders in the same bottom organic cover (Mavuti and Litterick 1991). Insects, gastropods and worms are reported to be more tolerant to high organic pollution (Ngupula et al. 2012).

**Table 6:** Mean abundance (individuals/m^2^) of macro invertebrates species at the intermediate and along the cages encountered before the start of cage farming activities at Shirati bay

Microinvertebrates	Along the cage	Intermediate
Gastropods		
*Bellamya costulata*	69	52
Bivalves		
*Sphaerium nyansae*	95	651
*Sphaerium stuhlmanni*	139	104
*Caelatura alluaudi*	35	0
*Caelatura monceti*	52	130
Worms	43	26

**Table 7:** Mean abundance (individuals/m^2^) of macro invertebrates species at the intermediate and along the cages encountered after the start of cage farming activities at Shirati bay

Macroinvertebrates	Along the cages	Intermediate
Gastropods		
*Bellamya unicolor*	17	52
*Bellamya costulata*	121	104
*Melanoides tuberculata*	95	0
Bivalves		
*Sphaerium nyansae*	1293	78
*Sphaerium stuhlmanni*	382	156
*Caelatura alluaudi*	78	104
*Caelatura monceti*	121	52
Insects		
*Ephemeroptera*	0	0
*Heptageniidae*	35	0
Chaoborus larvae	35	26

**Table 8:** Shannon diversity index, species richness and equitability (evenness) of benthic macro invertebrate species within the cage areas and at the intermediate site recorded before and after the set of cages

	Along the cage		Intermediate	
	Before	After	Before	After
Richness	6	9	5	7
Diversity index	1.317	1.044	0.791	1.604
Equitability	0.735	0.475	0.491	0.825

## Conclusions

Despite the fact that our results indicated no consistent environmental changes at the cage sites. The localized water quality dynamics observed are a common phenomenon in cage culture and does not explain any environmental problem. Generally Lake Victoria is ranked as hyper-eutrophic lake (OECD 1982). Therefore, caution should be taken when authorizing cage culture in the lake so as not to compromise the already deteriorated water quality and impair the ability of the lake to provide benefits sustainably to the communities whose livelihood depend on it. Thus, we suggest a proper site selection and continuous environmental monitoring be an essential component when considering introduction of fish cage culture in Lake Victoria.
